# Contemporary Treatment of NMIBC—Is It Time to Move on from BCG?

**DOI:** 10.3390/jcm13144112

**Published:** 2024-07-14

**Authors:** Rachel Passarelli, Vignesh T. Packiam

**Affiliations:** 1 Division of Urology, Rutgers Robert Wood Johnson, New Brunswick, NJ 08901, USA; rp1169@rwjms.rutgers.edu; 2 Division of Urology, Rutgers Cancer Institute of New Jersey, New Brunswick, NJ 08901, USA

**Keywords:** non-muscle-invasive bladder cancer, BCG, intravesical chemotherapy, BCG alternatives

## Abstract

Non-muscle-invasive bladder cancer (NMIBC) encompasses approximately three-quarters of all bladder cancer (BC) diagnoses. Intravesical Bacillus Calmette-Guerin (BCG) has been the long-standing gold standard treatment for patients following endoscopic resection. However, despite reasonable efficacy, recurrence rates are still suboptimal, and this, combined with treatment tolerability and BCG shortages, has prompted an investigation into alternative treatment modalities. Advances in this landscape have been predominantly for patients with BCG-unresponsive disease, and there are currently four FDA-approved treatments for these patients. More recently, trials have emerged looking for alternatives to BCG for patients who are treatment-naïve. We performed a literature search via PubMed to find recent publications on alternatives to BCG, as well as a search on clinicaltrials.gov and recent conference presentations for ongoing clinical trials. Studies have shown that combination intravesical chemotherapy, combination intravesical therapy with BCG, and combination intravenous therapy with BCG preliminarily have good efficacy and safety profiles in this disease space. Ongoing trials are underway, and we anticipate as these studies mature, there will be a shift in NMIBC treatment regimens.

## 1. Introduction

Bladder cancer (BC) is predicted to be newly diagnosed in 83,000 patients in 2024 and is currently the fourth most diagnosed cancer in men [1]. Risk stratification is based on grade and stage with a specific distinction based on depth of invasion into the bladder. Non-muscle-invasive bladder cancer (NMIBC) encompasses nearly 75% of all BC cases [2]. Treatment for NMIBC consists of complete endoscopic resection followed by observation or adjuvant therapy, depending on risk stratification. Bacillus Calmette-Guerin (BCG) is the gold standard guideline recommended adjuvant treatment regimen for both intermediate-risk (IR) and high-risk (HR) NMIBC [3,4,5]. A long-standing treatment option for NMIBC, BCG, has been shown to be the optimal treatment for HR disease in terms of decreasing recurrence as well as progression [6,7].

Although BCG is efficacious, there are several contemporary problems associated with its use. First, relapse rates in NMIBC are high, with 20–40% of patients experiencing recurrence and treatment-refractory disease [8,9]. Heterogeneous results in the literature can be attributed to variations in clinicopathological characteristics between studies and discrepancies in treatment and surveillance schedules. Next, BCG has been shown to have significant patient tolerability limitations. Approximately 60–70% of patients will experience either local or systemic side effects from intravesical instillation, such as chemical cystitis, fatigue, body aches, or hematuria, and some patients will stop treatment as a result [10,11]. While most dose-limiting side effects are related to tolerability alone, BCG sepsis is a rare but life-threatening potential sequela. Most importantly, access to BCG remains problematic, with intermittent shortages prompting guideline modifications and the investigation of treatment alternatives [12,13].

The global BCG shortage can be attributed to both increasing demand and alterations or issues in the supply chain. There are multiple manufacturing companies, each producing a different strain of the virus. In the US, there is currently only one available and approved strain, TICE BCG, which is produced by Merck. In response to the 2019 shortage, The American Urological Association (AUA) released revised guideline statements with the aim to better allocate limited BCG to appropriate patients. Recommendations included alternative treatments for IR disease, dose reduction, decreasing maintenance regimens, and prioritization of available BCG for induction courses to those with HR disease [13]. Unfortunately, some studies have shown higher recurrence rates and total cost per patient during times of BCG shortage [14,15].

A combination of efficacy and tolerance issues, as well as the long-standing BCG shortage, has prompted an investigation into alternative treatments to BCG for patients with NMIBC. Herein, we discuss first-line BCG alternatives for NMIBC through a review of recent literature and current ongoing areas of exploration.

## 2. Materials and Methods

We performed a literature search via PubMed to find recent publications on first-line treatment options for patients with NMIBC aside from BCG. We also queried clinicaltrials.gov and searched for recent conference presentations of ongoing clinical trials. Keywords included NMIBC, BCG, BCG alternatives, intravesical chemotherapy, immunotherapy, and combination therapy. Studies were included or excluded based on the authors’ expertise. We performed a narrative review of the selected studies.

## 3. Discussion

### 3.1. Non-BCG First-Line Therapies for Treatment-Naïve NMIBC

#### 3.1.1. Intravesical Gemcitabine

Intravesical Gemcitabine has been evaluated in direct comparison to other treatment options for NMIBC. A Cochrane review including 1222 patients in total assessed intravesical Gemcitabine compared to placebo, Mitomycin C, and BCG. With respect to placebo, Gemcitabine may reduce the risk of recurrence, but in comparison to Mitomycin C, Gemcitabine may reduce both the risk of recurrence and progression. Finally, for the HR BCG-exposed recurrent NMIBC, Gemcitabine was found to reduce recurrence and progression rates compared to BCG, 41% versus 97% and 6% versus 33%, respectively. However, side effects and adverse event (AE) profiles between these treatment options are inconclusive [16].

#### 3.1.2. TAR-200

TAR-200, an indwelling device that elutes intravesical Gemcitabine over 3 weeks, is under investigation as an exciting novel drug delivery system. Results from a Phase 1 trial evaluating the effects of TAR-200 in 12 patients with recurrence after a prior diagnosis of IR NMIBC showed safety and efficacy, with a complete response(CR) of 42% at cut-off and no Grade 3 or higher AE [17]. There were compelling interim results of this agent recently reported in the BCG-unresponsive space [18]. TAR-200 is currently under evaluation for BCG-naïve patients in the SunRISe-3 trial, a 3-arm study assessing TAR-200 plus Cetrelimab vs. TAR-200 plus BCG vs. TAR-200 alone in patients with HR NMIBC (NCT05714202). The results of this study have not yet been reported. TAR-210 is a similar intravesical drug delivery system that provides local continuous release of Erdafitnib, a fibroblast growth factor receptor (FGFR) tyrosine kinase inhibitor, for 90 days. Preliminary studies have focused on the use of this device in patients with recurrent NMIBC with CR rates of 82% and 87% in BCG-refractory and IR disease, respectively, at the time of data presentation [19,20]. The Phase 3 MoonRISe-1 trial (NCT06319820) is currently underway, assessing TAR-210 vs. intravesical chemotherapy in patients with IR NMIBC and FGFR alterations.

#### 3.1.3. Mitomycin C

Mitomycin C(MMC) has also been extensively evaluated as an option for treating NMIBC. In direct comparison to Gemcitabine, MMC has been shown to have a slightly less favorable recurrence profile with a higher incidence of chemical cystitis- in one study, at median 36 months follow-up recurrence-free rates were 72% and 61% in Gemcitabine and Mitomycin C, respectively [21]. A Cochrane review comparing BCG to MMC in patients with IR and HR NMIBC found that BCG may reduce the time to recurrence compared to MMC. In terms of the progression of the disease, BCG was found to have little impact compared to MMC (HR 0.96, 95% CI 0.73 to 1.26) [22]. A comparative study of 89 patients with frequently recurrent Ta/T1 NMIBC receiving induction and maintenance BCG versus MMC suggested decreased recurrence incidence in the BCG cohort, 59.1% versus 80.0%, respectively [23]. Trials have investigated the idea of hyperthermic MMC or MMC instilled at higher temperatures. In randomized studies, hyperthermic MMC has been shown as a safe and effective treatment option comparable to or even slightly superior to BCG [24,25]. However, MMC can be associated with long-lasting side effects, such as chronic pelvic pain, severe lower urinary tract symptoms, or fistulas requiring reconstructive surgery or urinary diversion [26].

#### 3.1.4. Sequential Intravesical Gemcitabine and Docetaxel

Gemcitabine/Docetaxel (Gem/Doce) was initially investigated as a salvage treatment for BCG-unresponsive NMIBC. In 2015, Steinberg et al. retrospectively assessed treatment success, defined as the lack of recurrence and bladder preservation, in a cohort of patients predominately with recurrence after BCG. They noted treatment success to be 66% at first surveillance, 54% at 1 year, and 34% at two years. Of their cohort of 45 patients, 78% avoided cystectomy [27]. In a similar retrospective patient cohort with a longer duration of follow-up information available, progression-free survival(PFS) and cancer-specific survival(CSS) at 5 years were noted to be 82% and 91%, respectively, with a bladder preservation rate of 75% [28]. While currently utilized off-label, data in this landscape is promising, and Gem/Doce is currently applied in the real practice setting. In a 2022 survey with responses from 198 practicing urologists, 51(71%) reported prescribing Gem/Doce in their practice in the last year. Gem/Doce was most commonly administered for BCG-unresponsive NMIBC with or without carcinoma in situ(CIS) (67%), BCG-naïve HR NMIBC (25%), or primary treatment of IR NMIBC (15%) [29].

Due to the promising results of Gem/Doce in BCG-unresponsive patients, more recent studies have emerged and are currently underway assessing Gem/Doce in BCG-naïve patients. A retrospective review evaluated ten years’ worth of outcomes for 312 patients with HR NMIBC who either received Gem/Doce or BCG following TURBT. At 6, 12, and 24 months, recurrence-free survival (RFS) estimates in the BCG group were 76%, 71%, and 69%, respectively, compared to 92%, 85%, and 81% in the Gem/Doce group. On multivariable analysis, high-grade RFS and RFS were superior, HR 0.57 and 0.56, respectively, in the Gem/Doce cohort compared to BCG-treated patients. Furthermore, induction BCG was associated with more treatment discontinuation than Gem/Doce, 9.2% vs. 2.9% [30]. Gem/Doce has also been evaluated specifically in patients with IR disease. In a retrospective study of 77 patients with IR NMIBC treated with intravesical Gem/Doce, 2 year RFS was noted to be 71%. Of this cohort, 43% had received some prior intravesical therapy, and patients who were treatment-naïve prior to initiation were noted to have superior RFS compared to prior treated, 79%, and 64%, respectively. Gem/Doce was well tolerated, with only one Grade 3 AE and 96.1% tolerating a full induction regimen [31]. Results from a single-arm prospective Phase 2 study assessing the response to Gem/Doce in HR NMIBC BCG-naïve patients were recently published. This study out of Johns Hopkins showed that out of 25 patients, 3 month CR was 100% and RFS at 12 months was 92%, with two recurrences at 9 and 12 months in patients with initial HGT1. Although Grade 1 AE occurred in 92% of patients, only five experienced a Grade 3 or higher AE, which consisted of hematuria or urinary tract infection [32].

The BRIDGE trial, a Phase 3 trial comparing BCG vs. Gem/Doce for high-grade (HG) NMIBC, is currently recruiting and will aim to evaluate non-inferior event-free survival for Gem/Doce. A total of 870 patients will be stratified by the presence of CIS or CIS with papillary NMIBC and then randomized into two treatment arms: weekly Gem/Doce for 6 weeks followed by maintenance for 2 years or weekly BCG for 6 weeks followed by periodic 3 week maintenance. Key secondary objectives aim to assess bladder cancer-specific quality of life between these two agents [33].

### 3.2. Modified BCG First-Line Therapies for Treatment-Naïve NMIBC

There are three main approaches to modified BCG: (1) utilization of an alternate strain, (2) combination intravesical therapy with BCG, and (3) combination systemic therapy with BCG.

#### 3.2.1. Alternative BCG Strains

While the TICE strain of BCG is the only one currently available and approved in the US, there are efforts underway to approve additional strains. The S1602 study assessed three arms: patients who received intravesical TICE BCG, patients who received Toyko-172 BCG, and patients who received intradermal BCG followed by intravesical BCG with the Toyko-172 strain. The primary objectives are twofold: to show the non-inferiority of the Toyko-172 strain of BCG with regard to HG recurrence and to assess the effect on recurrence of priming with intradermal BCG vaccine prior to intravesical treatment. S1602 has completed accrual, and the results are impending [34].

#### 3.2.2. Combination Intravesical Therapy with BCG

There are several combination therapies that aim to augment the immunologic effect and efficacy of BCG. Nagapendekin Alpha-inbakicep (NAI) is an IL-15 super agonist that activates natural killer and T cells. Results from a recent trial leading to this treatment, FDA approval in April 2024 assessing the combination of BCG and NAI compared to NAI alone in patients with BCG-unresponsive CIS with or without papillary disease, suggest favorable results. Of 82 patients treated with combination therapy, CR was noted to be 55%, 56%, and 45% at 3, 6, and 12 months, respectively. Out of patients who had responded to treatment, 90% were able to avoid cystectomy. Only three Grade 3 immune-related AEs were noted [35]. This treatment regimen has also been preliminarily evaluated in patients with BCG-naïve disease. A Phase 1B trial evaluated the safety and tolerability of 6 weekly instillations of a combination of NAI and BCG in nine BCG-naïve HR NMIBC patients. At 2 years follow-up, all patients were noted to be disease-free, and all AE were below Grade 3 [36]. A long-term follow-up study for these patients is upcoming (NCT05981131), and Phase 2 of this trial is currently underway (NCT02138734).

#### 3.2.3. Combination Intravenous Therapy with BCG

The checkpoint inhibitors Pembrolizumab, Atezolizumab, and Durvalumab have all been assessed in combination with BCG for treatment-naïve disease.

Pembrolizumab, a PD-1 inhibitor, was initially approved for the treatment of BCG-unresponsive HR NMIBC in 2020 as the first alternative to RC in this cohort. This approval was based on the findings of the Phase 2 Keynote-057 trial, which examined the response to intravenous Pembrolizumab. Cohort A of the trial consisted of 96 patients with BCG-unresponsive CIS. At the 3 months rate, 41% of patients were noted to have CR, and at 12 months, this was maintained at 19% [37]. Results from Cohort B, those with papillary disease, suggest that out of 132 patients, 12 months disease-free survival (DFS) and PFS were noted to be 43.5% and 83%, respectively [38]. A major limitation of Pembrolizumab is its immune-related side effects, with 66% having any AE, of which 13% were Grade 3 or higher [37]. Despite its efficacy and approval, Pembrolizumab has been shown to be not cost-effective in this patient cohort when compared to RC or salvage intravesical chemotherapy [39].

Pembrolizumab is under investigation in combination with BCG as a first-line treatment option for patients with HR NMIBC. BCG and Pembrolizumab are both distinct immunotherapies that have proven effects in urothelial carcinoma (UC); thus, they may have synergistic activity acting together. There are two trials underway assessing BCG and Pembrolizumab in this fashion. One is a Phase 2 trial (NCT03504163) out of Memorial Sloan Kettering assessing BCG + Pembrolizumab as first-line treatment in patients with HR NMIBC with expected completion in 2025. A second Phase 3 trial, also expected to be complete in 2025, Keynote-676(NCT03711032), assesses the combination of BCG and Pembrolizumab in comparison to BCG alone for both patients with BCG-naïve and BCG recurrent disease.

Atezolizumab, an IgG1 monoclonal antibody targeting PD-L1, has been evaluated as an option for patients with HR BCG-unresponsive NMIBC. Despite having similar clinical efficacy to other immunotherapy treatment options in this patient cohort, results are evaluated cautiously given a relatively high rate of serious immune-related side effects (16%) that are also paralleled in other similar treatments [40]. Investigation of Atezolizumab in BCG-naïve NMIBC is also underway. BladderGATE (NCT04134000) is a Phase 1b-2 trial assessing the response to Atezolizumab and BCG in patients with HR NMIBC who are BCG-naïve or have not received BCG in 2 years. Patients are to receive Atezolizumab and then either full dose or half dose BCG for induction and then maintenance courses with the aim to assess dose-limiting toxicity [41]. Interim analysis of 34 patients showed no dose-limiting toxicity(DLT), and the treatment regimen has been deemed overall tolerable [42]. Preliminary efficacy results of 36 patients suggest a 2 year DFS of 72.8%, local recurrence rate of 14%, and 8% progression to local MIBC. There were seven patients who discontinued Atezolizumab secondary to immune-related AEs [43]. ALBAN(NCT03799835) is a Phase 3 trial out of France evaluating the efficacy and safety of Atezolizumab and BCG compared to BCG alone in patients with BCG-naïve HR NMIBC. This trial began in 2018 and aims to be completed by 2028 [44].

Durvalumab, another PD-L1 inhibitor, is currently undergoing investigation in the Phase 3 POTOMOC trial (NCT03528694) assessing the response in patients with HR BCG-naïve NMIBC. The goal is to enroll approximately 1000 patients with primary endpoint DFS. The trial is comprised of three treatment arms: BCG induction + maintenance for 24 months, BCG induction + maintenance + Durvalumab, and BCG induction + Durvalumab. The estimated completion for this trial is in 2025 [45].

### 3.3. BCG-Unresponsive Therapies for Future Assessment as First-Line Therapies for Treatment-Naïve NMIBC

#### 3.3.1. BCG-Unresponsive Definition

In 2018, the FDA released guidelines to better define at what point disease can be considered BCG unresponsive. Criteria include HG T1 disease present at first cystoscopic evaluation after solely induction BCG, recurrent HG Ta/T1 that presents within 6 months of completing an adequate BCG therapy course, or persistent or recurrent carcinoma in situ (CIS) with or without HG Ta/T1 disease that presents within 12 months of completion of BCG therapy [46,47]. BCG-unresponsive disease is based on the cornerstone of adequate BCG courses, which entails at least 5 of 6 induction doses and at least 2 of 3 maintenance or 2 of 6 s induction doses [46]. Current guidelines maintain that the treatment for BCG-unresponsive disease is radical cystectomy (RC) [3]. Given the high morbidity associated with this surgery, many patients are not ideal candidates or may strongly prefer bladder preservation strategies.

#### 3.3.2. Potential Future Trials

There are currently four FDA-approved pharmacologic alternatives to RC for BCG-unresponsive patients: intravesical Valrubicin, systemic Pembrolizumab (Keytruda), Nadofaragene Firadovenoc (Adstiladrin), and, most recently, Nogapendekin Alfa-inbakicept [48,49,50,51]. Adstiladrin was approved in 2022 after a Phase 3 RCT or 157 patients showed an anytime CR of 60% maintained in 30% at 1 year in patients with BCG-unresponsive CIS. Most importantly, this drug is delivered on a liberating 3 month dosing schedule and is extremely well tolerated, with only 4% of patients experiencing a Grade 3 or higher AE [52]. Cretostimogene grenadenorepvec (CG), an oncolytic adenovirus that targets retinoblastoma pathway deficient bladder tumor cells, has shown to be a safe and effective treatment in BCG-unresponsive NMIBC [53]. This option additionally is under investigation for patients with IR NMIBC(PIVOT-006); however, there are no trials specifically investigating these options in HR BCG-naïve patients. Erdafitinib is under investigation in the THOR-2 trial, which compares the response of patients with recurrent NMIBC with select FGFR mutations to either oral Erdafitinib or intravesical chemotherapy. Preliminary results suggest favorable outcomes for both HG and LG recurrent NMIBC [54,55]. Radiotherapy has also been investigated as an alternative treatment option for NMIBC. Early prospective studies have suggested reasonable PFS and cystectomy-free rates [56]. NCT06310369 is an upcoming EORTC Phase 2 trial assessing radiotherapy combined with a radiosensitizer for patients with BCG-unresponsive NMIBC [57]. We suspect that as these agents and techniques are approved and utilized in the treatment-refractory setting, they may also be investigated in the first-line treatment-naïve setting.

### 3.4. Treatment-Unresponsive NMIBC: A Future Definition Agnostic to BCG?

As drug shortages continue, urologists will continue to turn to effective and available alternative treatments to BCG. Furthermore, the BRIDGE trial may provide Level 1 evidence that Gem/Doce is non-inferior to BCG, raising the rationale for its use and adoption. In this future scenario, new definitions of NMIBC treatment may be necessary. For example, if BCG is not the initial treatment given to most patients, the terms BCG-exposed and BCG-unresponsive may become irrelevant. A more appropriate term may simply be treatment-unresponsive NMIBC, with either the number of prior treatments or time elapsed since initial treatment becoming more relevant than specific BCG-exposure status. There is some data to suggest that these variables have an independent prognostic effect on NMIBC [58,59,60].

## 4. Conclusions

BCG is the historical gold standard for NMIBC treatment; however, given the high recurrence rates, drug shortages, and dose-limiting side effects, there is an ongoing search for alternative treatment modalities. There has been significant growth in the approval of treatments for patients with BCG-unresponsive NMIBC, but fewer dedicated studies and formal alternative treatment approval for BCG-naïve patients; however, trials are now emerging in this sphere. While currently used off-label, Gem/Doce has preliminarily been shown as a safe and effective first-line alternative to BCG, with later-stage trials underway. As the paradigm begins to shift with the proven efficacy and accessibility of alternative treatments, we will have to change the way we approach recurrent disease, broadening our definition from BCG unresponsive to treatment-unresponsive. In this review, we report the recent advances in BCG alternative treatment for NMIBC through an evaluation of the current literature and ongoing trials.
